# Validity and reliability of the Clinical Learning Environment, Supervision and Nurse Teacher (CLES+T), Turkish version^1^

**DOI:** 10.1590/1518-8345.2413.3037

**Published:** 2018-09-06

**Authors:** Selma Atay, Fatma Yılmaz Kurt, Gülbahar Korkmaz Aslan, Mikko Saarikoski, Hilal Yılmaz, Volkan Ekinci

**Affiliations:** 2 PhD, Assistant Professor, Fundamental Nursing Department, Çanakkale Onsekiz Mart University, School of Health, Çanakkale, Turkey.; 3 PhD, Assistant Professor, Pediatric Nursing Department, Çanakkale Onsekiz Mart University, School of Health, Çanakkale, Turkey.; 4 PhD, Assistant Professor, Department of Public Health Nursing, Pamukkale University, Pamukkale, Turkey.; 5 PhD, Associated Professor, Department of Nursing Science, University of Turku, Turku, Finland.; 6 Undergraduate student in Nursing, Çanakkale Onsekiz Mart University, School of Health, Çanakkale, Turkey.

**Keywords:** Nursing Student, Clinical Environment, Scale, Satisfaction, Validity, Reliability

## Abstract

**Aim:**

A methodological type of study was conducted for the purpose of investigating the validity and reliability of the Turkish version of the Clinical Learning Environment, Supervision and Nurse Teacher **(**CLES+T) evaluation scale of the clinical learning environment of students, clinical nurses, and educators.

**Methods:**

Sample was comprised of 602 Turkish nursing students with clinical practice experience at the hospital. The CLES+T, developed by Saarikoski, was used for data collection. Language equivalency, internal consistency, item-total correlation, and structure validity were conducted within the scope of the validity and reliability study on the CLES +T scale.

**Results:**

It was determined that item-total correlations of four items were lower than 0.30, and those items were removed from the scale as a result of item analysis. The Cronbach’s alpha value of the scale was 0.93-0.99; item total point correlations of the scale varied between 0.45 and 0.66; six factors were identified in the CLES+T factor analysis study, with a total variance explained by these six factors of 64%.

**Conclusion:**

According to the findings of the research, the CLES+T Turkish version was found to be a valid and reliable scale, which can be used to evaluate satisfaction of nursing students with their clinical education in Turkey.

## Introduction

Clinical education is a process that provides the student with the opportunity to practice his/her theoretical knowledge, gain professional identity, and learn by practice; thus, it is crucial in nursing education programs^1^. Clinical practice fields enable the students to combine their cognitive, psychomotor, and affection skills and contribute to the development of these competencies^2^. In order for the students to be able to benefit from these opportunities, clinical learning environments must be designed in a way that serve these ends, and the students must be supported.

Clinical learning and clinical learning environments have been subjects of research since 1990^3^. The clinical learning environment plays a crucial role, especially in the clinical education of nursing students^4^ ^-^ ^6^. The clinical learning environment includes attributes of the clinical work setting which nurses perceive to inﬂuence their professional development^7^. Employee and student relationships and significant learning situations in the clinical learning environment constitute the pedagogical atmosphere of the clinic^8^. Good relationships between individuals, support, and feedback affect the clinical learning environment, and are important for positive learning^9^ ^-^ ^10^. Numerous studies emphasize that the clinical environment is crucial in learning and learning outcomes^8^ ^,^ ^11^. One study discovered that a supportive learning environment creates a significant difference in students’ learning. The pedagogical atmosphere of the service affects the learning process and competencies. It has been emphasized that the skills of problem solving and asking questions would develop in a positive pedagogical atmosphere^3^ ^,^ ^12^ ^-^ ^13^. A collaborative leadership style, less hierarchical structure, and positive team spirit allow nursing students to feel that they are supported in uncertainties^3^ ^,^ ^6^. The acceptance of nursing students as “team members” in the clinical environment, and consideration of student opinions and experiences in the solution of problems, contributes to their professional development^14^. This critical thinking and mutually innovative atmosphere may influence nursing care and quality, thus it would also be reflected in the patient-nurse relationships^14^.

The learning environment is also related to the psychosocial environment of the health service. The most important feature of a good learning environment is the presence of trust from the perspective of the student. A just environment is possible by seeing the students as part of the problem solving process, and improving the culture of tolerance for mistakes^3^ ^,^ ^15^. 

During the period of clinical education, which is the basic part of nursing education, nurse educators especially are essential factors. Competency of nurse educators is the most important factor that determines the quality of the education. For this reason, nurse educators play a crucial role in both education and clinical practice^16^. Therefore, having nurse educators who are well-equipped, positive role models, with awareness and experience, is important in order to achieve practice purposes^17^. Numerous studies indicate that students who spend their clinical education with experienced and professional teaching staff and nurses adjust more easily to the clinic, develop a better concept of the professional role^18^, develop critical thinking abilities, have improved self-sufficiency,^18^ and communication skills^19^. Additionally, research emphasizes that the collaboration between educators and clinical nurses is also important in a good clinical learning environment^14^ ^,^ ^20^ ^-^ ^21^. Nurse educators and clinical nurses are the primary responsible agents for different learning experiences^16^ ^,^ ^22^. 

It has been stated that clinical nurse supervision is also crucial during the clinical practice process in student competency^23^ ^-^ ^24^. The concept of the clinical nurse has been used in the meaning of unifying and supporting nursing students. For example, they are people who teach and evaluate practice skills, complement the clinical knowledge of nursing students, provide feedback, help them to perform analysis between theory and practice, are a role model, and in addition, help students to socialize. According to Löfmark and Wikblad, negative attitudes and behaviors of clinical nurses affects the learning process of nursing students. There is evidence regarding the exact importance of one-to-one education for the learning and development of students in clinical practice^23^. Generally, the clinical nurse is responsible for the supervision of the students. Similarly, whether the service culture is negative or positive reflects the leadership style of the responsible clinical nurse. A positive team spirit and less hierarchical leadership may enable nursing care, motivation of the personnel, and supervision of the students^25^. In their studies, Lofmark and Wikblad stated that attributing responsibility, independence, providing opportunity for different tasks, and giving feedback are among the factors that make students’ learning easier, whereas supervision and insufficient opportunities are the factors that hinder learning.

The importance of clinical practice in converting theoretical knowledge into skills, and the development of professional identities of the students in nursing education cannot be overlooked. The evaluation of the clinical environment, clinical nurses, and educators, which are essential in the development of professional identities of the students, is very important. To this aim, this study was conducted to determine the validity and reliability of the Turkish version of the CLES+T scale.

## Method

This study has been conducted methodologically in order to test the validity and reliability of the Turkish version of the CLES +T scale.

Sample: The research population was comprised of the students studying in the nursing department of a university. The criterion of selecting a minimum of five people for each scale item was used to determine sample size^26^. As the CLES+T scale is comprised of 34 items in total, 602 students were used for the scope of sampling. The prerequisite of having performed clinical practice at least for one term at the hospital was among the sampling inclusion criteria. Data was collected in the 2015-2016-spring semester. The data tool was administered to the students in the classroom environment by a researcher, at the end of the clinical practice. The time required to complete the form was approximately 20 minutes.

In the study, the CLES+T scale was used as the data collection tool, originally developed by Saarikoski and Leino-Kilpi in 2002, and revised in 2008. The CLES+T scale evaluates the pedagogical atmosphere of the service, clinical educators, management style of the responsible nurse of the service, and the nursing care in the service. It is a 5-point Likert scale, comprised of 34 items in total (Completely disagree = 1, Disagree = 2, Partially agree / Partially disagree =3, Agree =4, Completely Agree =5). The original scale is comprised of five factors, namely: supervisory relationship (factor 1), pedagogical atmosphere on the ward (factor 2), role of the nurse teacher (factor 3), leadership style of the ward manager (factor 4), and premises of nursing on the ward (factor 5)^27^. We also collected demographic data (age, gender) and clinical data (unit type, length of clinical placement). 

 Within the scope of the validity and reliability study of the CLES+T scale, language equivalency, structural validity, and reliability studies were conducted. For the adaptation of the English form of the scale into Turkish, a translation-back translation method suggested in the literature and commonly accepted for adaptation was used.^28^ To this aim, firstly the original scale was translated into Turkish by two professional translators. The form translated into Turkish was examined by the researcher and a faculty member with a good command of English, then the best translation for each item was adopted. Following this stage, it was translated back into English by a professional Turkish language expert. Then the items in the original scale were compared to those in the back-translated scale, and meaning equivalency was ensured^28^ ^-^ ^29^. 

 Structural validity indicates the capacity of the scale to measure the entire concept or conceptual structure*.* Structural validity of the scale was evaluated by using confirmative factor analysis. In the study, for the prediction of the factor analysis, the criteria of having an eigenvalue of >1, a factor load of at least 0.40, and variance exploration rate to be 0.40 or greater used ^(^ ^28^ ^-^ ^29^. Barlett’s test is a statistical method used for controlling whether the data comes from a multivariable normal distribution. The significance of the chi-square test statistics, obtained as a result of this test, indicates that the data comes from a multivariable normal distribution^30^.

This is the capability of a measuring tool to provide consistent and stable measuring results. For the reliability of the scale, internal consistency and item total correlation analysis were used in the study. To assess internal consistency, Cronbach’s alpha was computed. Depending on the relevant literature, a minimum Cronbach’s alpha of > 0.70 is considered satisfactory^30^.

Item analysis is a correlation analysis that expresses the relation between the value each item takes within the measuring tool and the total value obtained from the entire measuring tool. The higher the correlation coefficient, the higher the relationship of that item to the quality to be measured. In the evaluation of total item correlation, items with a value >0.30 are considered satisfactory^29^. An item indicating a lower relationship with regard to total points implies that the item measures a different quality than the other items in the scale, and thus it is not reliable; such an item is removed from the scale.

Data was evaluated by computer using descriptive statistics for the demographic qualities of the sampling group. Varimax rotation and exploratory factor analysis (principal component analysis) were conducted for structural validity. The appropriateness of the data for factor analysis was examined using the Kaiser Meyer-Olkin (KMO) value and the Barlett’s test. The Cronbach’s alpha coefficient was calculated for internal consistency. A Pearson correlation analysis was conducted for item total point correlation.

Prior to the initiation of the study, written consent from Saarikoski was obtained for the use and adaptation of CLES+T to Turkish society. Ethical compliance for the study was obtained from the Medical Faculty Ethics Committee, under decision No.2015-13 on August 5, 2015. Permission was obtained in writing from members of the university administration to conduct the study. The principle of voluntariness was taken as a basis, and the student nurses comprising the sample group were informed about what was expected from them and their legal rights, and their consent was obtained.

## Results

There were 56.6% of the students who were in their third year; 79.9% of them were female, 36.0% of them had their apprenticeship experience within the internal medicine services, and 49.2% of them stated their time at the clinic had been four weeks. Additionally, the average age of the students was 20.5±1.5, and their average transcript grade was 2.60 ±0.4.

Factor analysis revealed that sample adequacy was confirmed by means of the Kaiser-Meyer-Olkin (KMO) test and Barlett’s test of sphericity. The KMO value of 0.940, and Barlett’s test (X^2^= 9772,44, p= .000) were found to be statistically significant.

As a result of item analysis, it was determined that item-total correlations of four items (10, 12, 13, 14) were <0.30, and those items were removed from the scale which then totaled 30 items and was composed of six subscales. We have shown this study, and the study of Saarikoski’s (2008) item subscales, in Table 1.

**Table 1:** Factors and the item of subscales of Saarikoski et al. (2008), and according to the study conducted in Canakkale, Turkey, 2015

İtem	Saarikoski et al. (2008)	Canakkale, Turkey study
Factor 1	Supervisory Relationship (1-8)	Supervisory Relationship (1-9)
Factor 2	Pedagogical atmosphere on the ward (9-17)	Pedagogical atmosphere on the ward (15-17)
Factor 3	Role of nurse teacher (18-26)	Role of nurse teacher (11, 24-26)
Factor 4	Leadership style of the ward manager (27-30)	Leadership style of the ward manager (27-29)
Factor 5	Premises of nursing on the ward (31-34)	Leadership style of the ward manager (30-34)
Factor 6		Relationship between student, mentor, and nurse teacher (18-23)

The scale of Saarikoski et al. (2008) and Johansson et al. (2010) has five subscales, but our scale identifies six subscales. Items 18-23 were part of the third factor in Saarikoski et al. (2008) and Johansson et al. (2010), but in our study these created their own factor. These items were part of the role of nurse teacher factor in Saarikoski’s scale, but in our scale these items are named as “Relationship between student, mentor, and nurse teacher”. Thus, in our study, factor 6 was named “Relationship between student, mentor, and nurse teacher”. The total variance explained by the six factors was 64%. Factor 1 accounted for 18% of response variance, factor 2 for 14%, factor 3 for 10%, factor 4 for 8%, factor 5 for 7%, and factor 6 for 7%.

**Figure 1:**
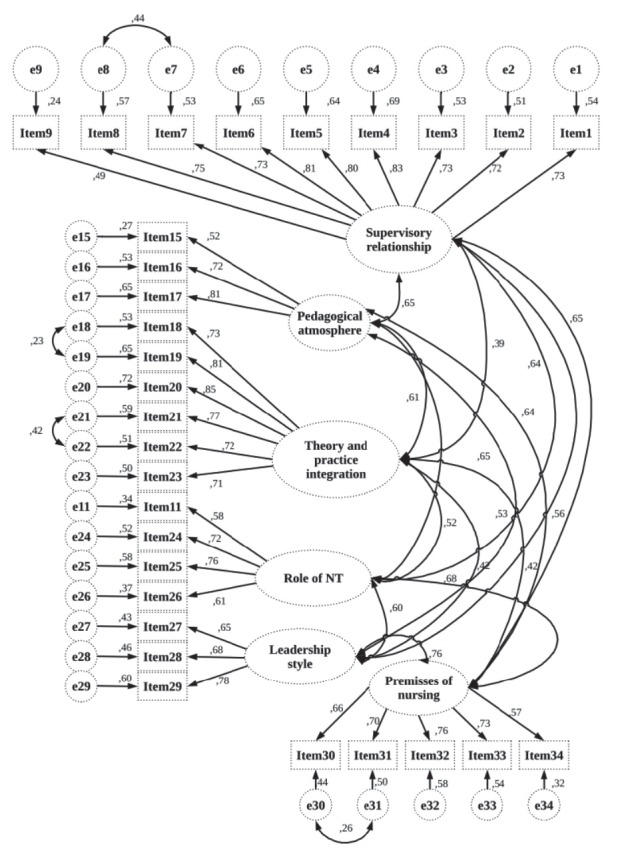
Model for 34 items of the Turkish version of CLES+T scale. Canakkale, Turkey, 2015

Figure 1 shows the confirmatory factor analysis (CFA) fit indexes of the two models. Through the two proposed models, these differences were studied. Supervisory relationship (factor 1: items 1-9), pedagogical atmosphere on the ward (factor 2: items 15-17), role of nurse teacher (factor 3: items 11, 24-26), leadership style of the ward manager (factor 4: items 27-29), premises of nursing on the ward (factor 5: items 30-34), and role of theory and practice integration (factor 6: items 18-23) were items in model.

Reliability results of the CLES+T scale are shown in Table 2. According to this, for 30 items the Cronbach’s alpha value is 0.93, and the alpha value in sub-dimensions varied between 0.70 and 0.76 When the relationship between the points of each sub-dimension and the total scale points was examined, reliability coefficients were found to be 0.93-0.96 The item means ranged between 2.57 and 3.68 (on a scale of 1-6). According to these findings, the total item point correlations of the scale ranged between 0.45 and 0.66 (Table 2).

**Table 2:** Statistics and Cronbach’s alpha coefficients for Factors 1-6 of the CLES+T, Turkish version (n=602). Canakkale, Turkey, 2015

	Mean CLES+T Turkish version *	SD^†^	Corrected item-total correlation	Cronbach’s alpha if item deleted
Supervisory relationship (α=.70)				
1 My supervisor showed a positive attitude towards supervision	3.26	.99	.62	.94
2 I felt that I received individual supervision	2.80	1.00	.56	.93
3 I continuously received feedback from my supervisor	3.02	1.03	.60	.93
4 Overall I am satisfied with the supervision I received	2.97	.98	.65	.94
5 The supervision was based on a relationship of equality	2.94	1.03	.62	.99
6 There was a mutual interaction in the supervisory relationship	3.16	.96	.66	.93
7 Mutual respect and approval prevailed in the supervisory relationship	3.21	1.02	.63	.93
8. The supervisory relationship was characterized by a sense of trust	3.10	.99	.66	.96
9. The staffs were easy to approach	3.22	.99	.49	.93
Pedagogical atmosphere on the ward (α=.76)				
15. There were sufficient meaningful learning situations on the ward	3.37	.86	.46	.93
16. The learning situations were multi-dimensional in terms of content	3.02	.96	.54	.94
17. The ward can be regarded as a good learning environment	3.23	1.01	.62	.94
Role of nurse teacher (α=.74)				
18. In my opinion, the nurse teacher was capable of integrating theoretical knowledge and everyday practice of nursing	3.66	1.00	.51	.93
19. The nurse teacher was capable of operationalizing the learning goals of this placement	3.63	.97	.52	.95
20. The nurse teacher helped me to reduce the theory-practice gap	3.60	.98	.51	.93
21. The nurse teacher was like a member of the nursing team	3.43	1.07	.49	.95
22. The nurse teacher was able to give his or her expertise to the clinical team	3.45	1.03	.43	.93
23. The nurse teacher and the clinical team worked together	3.34	.96	.65	.93
Relationship among student, mentor and nurse teacher (α=.75)				
11. During staff meetings (e.g. before shifts) I felt comfortable taking part in the discussions	2.76	1.12	.49	.93
24. The common meetings between myself, mentor and nurse teacher were comfortable experience	3.00	1.02	.57	.96
25. In our common meetings I felt that we are colleagues	2.57	1.06	.59	.93
26. Focus on the meetings was in my learning needs	3.17	.97	.48	.99
Leadership style of the ward manager (WM^‡^) (α=.76)				
27. The WM^‡^ regarded the staff on her/his ward as a key resource	3.06	1.04	.49	.93
28 The WM^‡^ was a team member	3.38	.97	.45	.97
29. Feedback from the WM^‡^ could easily be considered a learning situation	3.13	.98	.57	.94
Premises of nursing on the ward (α=.74)				
30. The effort of individual employees was appreciated	2.96	.97	.57	.93
31. The wards nursing philosophy was clearly defined	2.75	.97	.55	.93
32. Patients received individual nursing care	2.99	1.02	.57	.96
33. There were no problems in the information flow related to patients’ care	3.02	.97	.57	.93
34. Documentation of nursing (e.g. nursing plans, daily recording of nursing procedures etc.) was clear	3.68	.99	.48	.98

* CLES+T* - Clinical learning environment, supervision and nurse teacher; ^†^ SD- Standard deviation^; ‡^ WM - Ward manager

## Discussion

The Kaiser-Meyer-Olkin (KMO) value = 0.940 and the Barlett’s test (X^2^= 9772, 44, p= .000) were found to be of a significant level for the scale’s structural validity. Johansson et. al. found KMO= 0.93 and p<0.001 in their study. 

The total variance explained by the six factors was 64%. Factor 1 accounted for 18% of responses variance, factor 2 for 14%, factor 3 for 10%, factor 4 for 8%, factor 5 for 7%, and factor 6 for 7%. The variance explained in the study by Johansson et al., was 60.2% in a 34-item scale with 5 sub factors. In their study, Saarikoski et al. (2008) found a total explanation percentage of 67 of the sub-scale version, and Saarikoski and Leino-Kilpi (2002) found the explanation percentage of 64^25^. 

We tried to justify the reasons for the differences in the factor loadings, by conducting a CFA analysis. This indicates a suitable model fit for Model 1. An adequate fit to the data was suggested by values of X^2^/DF, IFI, CFI and RMSEA, with the exception of GFI. On the other hand, our data did not fit Model 2, which reproduced the conceptual structure of the original version of the CLES+T^27^. 

According to research findings, the total coefficient of the scale and the Cronbach’s alpha coefficient of the sub-scales are within an acceptable range. The Cronbach’s alpha coefficient is stated as 0.90 and as 0.96-0.77 for sub-scales in the findings of the study for the development of the original scale^27^. In the study by Johansson et al. (2010), the Cronbach’s alpha coefficient was 0.95, and was 0.96-0.75 for the sub-scales^20^ ^).^ In another study conducted in nine European countries, the Cronbach’s alpha coefficient was found to be between 0.96-0.83 for the sub-scales. As a result, we can conclude that the findings of our study are reliable, in consideration of the previous findings.

Finding item total point correlations of the scale between 0.45 and 0.66 demonstrates that item total point correlation values are at a reliable level. In the study by Johansson et al., item total correlation range of the scale varied between 0.35 and 0.91. In another study by Vizcaya-Moreno et al. (2015), for factors 1-5 , the corrected item-total correlation ranged from 0.36 to 0.92^31^.

## Conclusion

The CLES+T scale, the validity and reliability of which has been confirmed in the Turkish version, can be used in the evaluation of the satisfaction of student nurses with the clinical environment, clinical nurses, and nurse educators. This enables clinical education to be evaluated from the student’s perspective, and the quality of education can be improved.

Limitation of the study: The primary restriction of this research is the use of students from only two health colleges in the sampling.
